# Social Isolation, Loneliness and Subclinical Atherosclerosis

**DOI:** 10.1007/s11883-026-01447-7

**Published:** 2026-07-16

**Authors:** Huige Li, Ning Xia

**Affiliations:** 1 Institute of Pharmacology, University Medical Center, Johannes Gutenberg University, Mainz, 55131 Germany; 2 German Center for Cardiovascular Research (DZHK), Partner Site Rhine-Main, Mainz, Germany

**Keywords:** Social isolation, Loneliness, Subclinical atherosclerosis, Endothelial dysfunction, Cardiovascular disease, Vascular aging

## Abstract

**Purpose of Review:**

Social isolation and loneliness have emerged as important cardiovascular risk factors, yet the biological mechanisms linking adverse social experiences to cardiovascular disease remain incompletely understood. This review examines current evidence relating social isolation and loneliness to subclinical atherosclerosis and explores the underlying neuroendocrine, autonomic, inflammatory, and vascular mechanisms.

**Recent Findings:**

Recent epidemiological studies have associated social isolation and loneliness with endothelial dysfunction, arterial stiffness, carotid plaque, carotid intima-media thickness, and coronary artery calcium. Advances in transcriptomic and proteomic research have identified molecular signatures characterized by increased inflammatory signaling and altered immune regulation in socially isolated individuals. Experimental animal studies further support a causal relationship between adverse social environments and accelerated atherosclerosis.

**Summary:**

Accumulating evidence suggests that social isolation and loneliness contribute to subclinical vascular disease through interconnected pathways involving hypothalamic-pituitary-adrenal axis activation, autonomic dysregulation, chronic inflammation, oxidative stress, and endothelial dysfunction. Although available studies are limited and predominantly cross-sectional, findings support a biologically plausible link between social disconnection and early atherogenesis. Future longitudinal studies and intervention trials are needed to determine whether improving social connectedness can slow atherosclerosis progression and reduce cardiovascular risk.

## Introduction

The prevalence of loneliness and social isolation has increased substantially during recent decades. Demographic changes, population aging, delayed marriage, smaller household sizes, and changing patterns of social interaction have increased the number of individuals at risk for social disconnection [1–4]. Although loneliness is particularly common among older adults, it affects individuals across all stages of life [5, 6]. The growing prevalence of social isolation has stimulated increasing interest in its impact on physical health, particularly cardiovascular disease. While associations between loneliness, social isolation, and cardiovascular events are now well established, the biological pathways linking social disconnection to cardiovascular disease remain incompletely understood. Emerging evidence suggests that adverse social experiences may contribute to endothelial dysfunction, vascular aging, and the development of subclinical atherosclerosis, providing a potential mechanistic bridge between social disconnection and future cardiovascular events.

Because atherosclerosis develops over decades before the onset of clinical symptoms, the study of subclinical vascular disease offers a unique opportunity to investigate the early biological consequences of social isolation and loneliness. Imaging markers such as carotid intima-media thickness (IMT), carotid plaque, coronary artery calcium (CAC), and measures of vascular stiffness may provide important insights into the mechanisms through which social experiences become biologically embedded and influence cardiovascular risk.

The objective of this narrative review is to summarize current evidence linking social isolation and loneliness with subclinical atherosclerosis. Specifically, we review epidemiological studies examining associations between adverse social experiences and imaging markers of early vascular disease, discuss the biological mechanisms through which social disconnection may promote atherogenesis and highlight current knowledge gaps and future research directions.

## Methodology

This article is a narrative review of the literature. The literature search was conducted primarily in the PubMed database using multiple combinations of keywords related to loneliness, social isolation, social support, cardiovascular disease, and atherosclerosis, together with specific terms for subclinical atherosclerosis, including carotid intima-media thickness, carotid plaque, and coronary artery calcium. No restrictions on publication year were applied. Additional relevant articles were identified through backward and forward citation searching of selected publications. Articles were included based on their relevance to the topic and the authors’ judgment regarding their contribution to understanding the relationship between social isolation, loneliness, and subclinical atherosclerosis.

## Social Isolation and Loneliness as Cardiovascular Risk Factors

Psychosocial stress is increasingly recognized as an important cardiovascular risk factor. The INTERHEART study identified psychosocial stress as one of the leading modifiable risk factors for myocardial infarction worldwide, ranking behind only dyslipidemia and smoking [7, 8]. Because loneliness and social isolation represent common forms of chronic psychosocial stress [9, 10], considerable attention has focused on their role in cardiovascular disease development.

A growing body of epidemiological evidence indicates that poor social relationships are associated with increased morbidity and mortality. A meta-analysis involving more than 300,000 individuals demonstrated that strong social relationships increase the likelihood of survival by approximately 50% compared with weaker social relationships [11]. Subsequently, loneliness, social isolation, and living alone have all been associated with increased mortality risk [12, 13]. In addition, systematic reviews and meta-analyses have demonstrated that social isolation and loneliness are associated with increased risks of coronary heart disease and stroke [14]. Collectively, these findings support the recognition of social isolation and loneliness as independent cardiovascular risk factors [15].

More recent studies have strengthened this association. Recent meta-analyses confirmed that both social isolation and loneliness are associated with increased risks of cardiovascular disease incidence and cardiovascular mortality, even after adjustment for conventional cardiovascular risk factors [16]. These findings have recently been reinforced in large prospective population cohorts from Asia, where both social isolation and loneliness independently predicted incident cardiovascular disease [17]. Similarly, a systematic review focusing on individuals with established cardiovascular disease reported consistent associations between social disconnection and adverse cardiovascular outcomes [18]. The importance of social relationships for cardiovascular health has also been emphasized by the American Heart Association Scientific Statement on objective and perceived social isolation, which identified social disconnection as an emerging cardiovascular risk factor affecting both cardiovascular and brain health [19].

Although the association between social isolation, loneliness, and clinical cardiovascular disease is now well established, the mechanisms underlying this relationship remain incompletely understood. One possibility is that adverse social experiences contribute to the development of vascular dysfunction and subclinical atherosclerosis long before the onset of overt cardiovascular disease. This hypothesis is supported by growing evidence linking social disconnection to endothelial dysfunction, vascular aging, and imaging markers of subclinical atherosclerosis, which are discussed in the following sections.

## Biological Mechanisms Linking Social Isolation and Loneliness to Subclinical Atherosclerosis

**Fig. 1:**
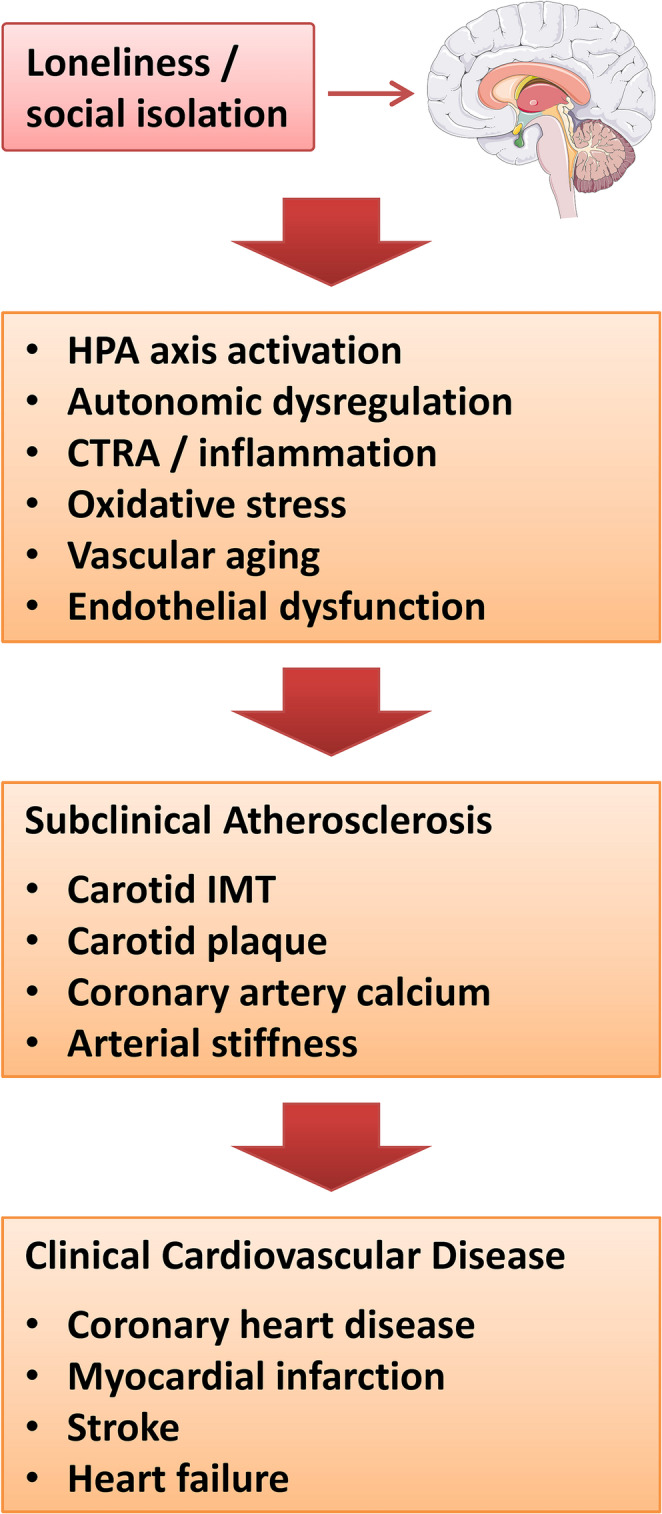
Proposed biological pathways linking social isolation and loneliness to subclinical atherosclerosis and cardiovascular disease. Social isolation and loneliness activate neuroendocrine and autonomic stress pathways, resulting in chronic inflammation, oxidative stress, and endothelial dysfunction. These alterations promote the development of subclinical atherosclerosis, including carotid plaque, carotid intima-media thickening, and coronary artery calcification, as well as vascular aging and arterial stiffening. Progressive vascular injury may ultimately lead to clinical cardiovascular disease, including coronary heart disease, stroke, heart failure, and cardiovascular mortality. Social support and other psychosocial factors may modify these relationships. CTRA, conserved transcriptional response to adversity; HPA, hypothalamic-pituitary-adrenal; IMT, intima-media thickness. The pictures of brain is from Servier Medical Art (https://smart.servier.com/) licensed under CC BY 4.0

Although the association between social isolation, loneliness, and cardiovascular disease is now well established, the biological mechanisms underlying this relationship remain incompletely understood. Current evidence suggests that adverse social experiences activate a network of interconnected neuroendocrine, autonomic, inflammatory, and vascular pathways that promote endothelial dysfunction and atherogenesis (Fig. 1). These biological responses may provide a mechanistic bridge between social disconnection and the development of subclinical vascular disease long before the occurrence of overt cardiovascular events.

### Neuroendocrine Stress Responses

Social isolation and loneliness are widely regarded as forms of chronic psychosocial stress. Among the biological systems activated by chronic stress, the hypothalamic-pituitary-adrenal (HPA) axis has received considerable attention. Activation of the HPA axis is one of the most consistent biological findings in lonely individuals [20]. The HPA axis regulates the production of glucocorticoids, including cortisol in humans and corticosterone in rodents, which exert important effects on metabolism, immunity, and cardiovascular function [21, 22].

Several studies have demonstrated enhanced HPA-axis activity in lonely individuals. Loneliness is associated with an exaggerated cortisol awakening response [23], elevated circulating cortisol concentrations [24, 25], and reduced glucocorticoid receptor sensitivity [26, 27]. These findings suggest that chronic social isolation is accompanied not only by increased glucocorticoid production but also by impaired glucocorticoid signaling.

Evidence from animal models supports a causal relationship between social isolation and HPA-axis activation. Prairie voles, which form stable social bonds, exhibit increased corticosterone concentrations following chronic separation from a bonded partner [28–30]. Interestingly, separation from a low-preference social partner does not produce comparable endocrine responses [28], suggesting that the perceived loss of social attachment rather than physical isolation per se may be critical.

Persistent activation of the HPA axis may contribute to atherogenesis through several mechanisms. Glucocorticoids potentiate the vasoconstrictor actions of catecholamines [31], impair endothelial nitric oxide synthase (eNOS) expression and activity [32, 33], and promote endothelial dysfunction. Since endothelial nitric oxide is a major anti-atherosclerotic and antihypertensive mediator [34, 35], chronic disturbances of glucocorticoid signaling may contribute to both vascular dysfunction and atherosclerosis. Furthermore, prolonged social stress may induce glucocorticoid resistance, thereby reducing the anti-inflammatory actions of endogenous glucocorticoids and favoring chronic vascular inflammation [36, 37].

Collectively, these findings suggest that chronic activation of stress-responsive neuroendocrine pathways may contribute to endothelial dysfunction, vascular inflammation, and early atherogenesis.

### Autonomic Dysregulation

In addition to HPA-axis activation, loneliness and social isolation alter autonomic nervous system function. The autonomic nervous system maintains cardiovascular homeostasis through a balance between sympathetic and parasympathetic activity. Chronic psychosocial stress shifts this balance toward sympathetic predominance, a state that favors hypertension, inflammation, endothelial dysfunction, and atherosclerosis.

Compared with the evidence for HPA-axis activation, studies examining sympathetic activation in loneliness have yielded less consistent results [21]. Nevertheless, socially isolated humans and non-human primates exhibit increased urinary concentrations of norepinephrine metabolites [25], suggesting sustained activation of sympathetic pathways. Importantly, social stress appears to exert stronger effects on local sympathetic signaling within tissues than on circulating catecholamine concentrations. Elevated tissue norepinephrine levels have been reported in socially stressed individuals despite minimal changes in plasma catecholamines [38, 39]. Such local sympathetic activation may have important consequences for inflammation and vascular biology.

An equally important aspect of autonomic dysregulation is impaired parasympathetic control. High-frequency heart rate variability (HF-HRV), a marker of vagal activity, is reduced in individuals with poor social integration and loneliness [40, 41]. Marriage and positive social relationships are associated with higher HF-HRV [42, 43], whereas social isolation in prairie voles reduces HF-HRV both at baseline and during stress exposure [44, 45]. Because reduced HF-HRV predicts cardiovascular morbidity and mortality [46–49], impaired vagal regulation may represent an important pathway linking social isolation to cardiovascular disease.

Oxytocin may play a role in this process. Administration of oxytocin improves autonomic regulation in socially isolated prairie voles [45] and enhances parasympathetic cardiac control in humans [50]. However, loneliness attenuates these beneficial autonomic responses [50], suggesting that impaired social bonding may compromise endogenous mechanisms that normally buffer stress-induced cardiovascular activation. Emerging experimental evidence further supports a direct antiatherogenic role of social bonding. Using ApoE-deficient mice, Ko et al. demonstrated that stable social bonds attenuated atherosclerosis through an oxytocin-mediated brain-liver axis, whereas social isolation accelerated plaque development [51]. Mechanistically, oxytocin signaling modulated hepatic lipid metabolism and systemic inflammatory responses, providing compelling experimental evidence that social relationships can directly influence atherosclerosis through neuroimmune pathways [51].

Through its effects on vascular tone, blood pressure regulation, endothelial function, and inflammation, autonomic imbalance may represent an important pathway linking social isolation to subclinical atherosclerosis.

### Immune Activation, Inflammation, Oxidative Stress, and Emerging Molecular Signatures

Chronic low-grade inflammation is increasingly recognized as a central mechanism linking social isolation to cardiovascular disease. Social isolation and loneliness are associated with a characteristic leukocyte transcriptional profile termed the conserved transcriptional response to adversity (CTRA), characterized by increased expression of proinflammatory genes and reduced expression of genes involved in antiviral immunity [26, 52, 53].

Transcriptomic analyses have identified monocytes and other myeloid-lineage cells as major contributors to this response [54]. Both lonely humans and socially isolated macaques exhibit expansion of classical CD14^++^/CD16^−^ monocytes, a population characterized by enhanced inflammatory activity [25]. At the same time, leukocytes from socially isolated individuals display reduced glucocorticoid receptor expression and increased activity of proinflammatory transcription factors such as NF-κB [25, 26]. Consequently, inflammatory signaling remains elevated despite increased cortisol production.

This inflammatory phenotype has important implications for atherosclerosis. Monocytes and macrophages play central roles throughout the atherosclerotic process, from endothelial activation and leukocyte recruitment to plaque growth and destabilization. Increased numbers of proinflammatory monocytes, together with impaired glucocorticoid-mediated anti-inflammatory signaling, therefore provide a plausible mechanistic link between loneliness and accelerated atherogenesis [25]. Consistent with this concept, lower levels of social connectedness have been associated with increased circulating concentrations of inflammatory mediators including interleukin-6 [55].

Oxidative stress may further amplify these inflammatory processes. Experimental studies have demonstrated increased oxidative stress in both the central nervous system and the vasculature following social isolation [56–58]. In socially isolated Watanabe heritable hyperlipidemic rabbits, enhanced vascular NADPH oxidase activity was accompanied by accelerated atherosclerosis [58]. Oxidative stress may reduce nitric oxide bioavailability, promote endothelial dysfunction, and amplify vascular inflammation, thereby contributing to lesion development [35, 59, 60].

Although the precise causal relationships remain incompletely understood, current evidence suggests that chronic social isolation promotes a proatherogenic milieu characterized by neuroendocrine activation, autonomic imbalance, inflammation, and oxidative stress. These mechanisms are highly interconnected and may act synergistically to accelerate vascular dysfunction and atherosclerosis.

Chronic inflammation and oxidative stress are central drivers of atherogenesis and may therefore represent key mechanisms through which loneliness and social isolation become biologically embedded within the vasculature.

Recent advances in high-throughput molecular profiling have further strengthened the biological link between social disconnection and cardiometabolic disease. In a large UK Biobank study involving more than 42,000 participants, social isolation and loneliness were associated with distinct plasma proteomic signatures enriched for pathways related to inflammation, complement activation, and antiviral responses [61]. Notably, many of the identified proteins were prospectively associated with cardiovascular disease, stroke, and type 2 diabetes, and Mendelian randomization analyses suggested potential causal links between loneliness and several cardiometabolic disease-related proteins. These findings provide molecular evidence that adverse social experiences may become biologically embedded through pathways relevant to atherogenesis and cardiovascular disease.

Taken together, chronic inflammation, oxidative stress, and adverse molecular remodeling may create a proatherogenic environment that promotes vascular injury and accelerates the development of subclinical atherosclerosis.

### Biological Aging and Vascular Aging

Emerging evidence suggests that accelerated biological aging may represent an additional mechanism linking social isolation and loneliness with cardiovascular disease. In two large population-based cohorts, both loneliness and social isolation were independently associated with accelerated biological aging [62, 63]. Conversely, persistent social connectedness over time was associated with attenuated biological aging assessed using artificial intelligence-enabled electrocardiographic age, suggesting that long-term social relationships may preserve cardiovascular resilience [62, 63].

These observations are consistent with earlier evidence linking loneliness to shorter telomere length, immune dysregulation, and reduced parasympathetic activity, all recognized hallmarks of accelerated aging [64]. Recent conceptual models further propose that loneliness influences biological aging through a brain-heart axis involving chronic stress responses, autonomic imbalance, inflammation, and vascular dysfunction, thereby providing an integrative framework connecting adverse social experiences with cardiovascular and cognitive aging [65].

The systemic consequences of social disconnection may extend beyond cardiovascular aging. A recent UK Biobank analysis reported that social isolation and loneliness were associated with earlier menopause and increased mortality among women, supporting the concept that adverse social experiences influence multiple aging-related biological processes [66].

### Endothelial Dysfunction: A Potential Link Between Social Isolation and Atherosclerosis

Endothelial dysfunction represents one of the earliest detectable manifestations of vascular injury and may provide a critical mechanistic bridge between psychosocial adversity and structural vascular disease. The endothelium plays a central role in maintaining vascular homeostasis through regulation of vascular tone, inflammation, thrombosis, and barrier function.

Recent clinical evidence supports this hypothesis. In a cross-sectional cohort of 312 adults undergoing assessment of peripheral endothelial function, social isolation was independently associated with peripheral endothelial dysfunction, an early marker of atherosclerosis. Individuals with greater social isolation exhibited lower reactive hyperemia index values, and the association remained significant after adjustment for traditional cardiovascular risk factors, supporting endothelial dysfunction as a potential mechanism linking social isolation to increased cardiovascular risk [67].

A central mechanism underlying endothelial dysfunction is reduced nitric oxide (NO) bioavailability. Loneliness and social isolation activate the HPA axis, resulting in sustained elevations of glucocorticoids. Excess glucocorticoid signaling has been shown to suppress eNOS expression and activity [32, 33], thereby reducing endothelial NO production. Concurrently, chronic psychosocial stress promotes oxidative stress and the generation of reactive oxygen species, which rapidly scavenge NO and further diminish its bioavailability. Reduced NO signaling impairs endothelium-dependent vasodilation, promotes endothelial activation, facilitates leukocyte adhesion and vascular inflammation, and contributes to a pro-atherogenic vascular environment. Experimental studies have further demonstrated impaired endothelium-dependent vasodilation in socially isolated animal models, consistent with reduced endothelial NO production. Collectively, HPA-axis activation, glucocorticoid-mediated eNOS suppression, and oxidative stress provide biologically plausible mechanisms through which loneliness and social isolation may promote endothelial dysfunction and initiate the early stages of atherosclerosis.

Beyond reduced NO bioavailability, disruption of endothelial barrier integrity may represent an additional mechanism linking loneliness to vascular disease. In a community-based cohort, loneliness was associated with endothelial dysfunction and increased circulating levels of soluble vascular endothelial-cadherin, a key protein involved in maintaining endothelial barrier function. Complementary mechanistic studies showed that stress-related signaling involving epinephrine and TNF-α reduced VE-cadherin expression and impaired endothelial integrity through JAK/STAT-dependent pathways. Loss of VE-cadherin may increase endothelial permeability and facilitate leukocyte infiltration into the vascular wall, thereby promoting vascular inflammation and early atherogenesis. These findings suggest that loneliness may contribute to atherosclerosis through both impaired endothelial signaling and structural disruption of the endothelial barrier [68].

Taken together, endothelial dysfunction may represent a critical intermediate phenotype linking social isolation and loneliness to structural vascular disease. By impairing NO signaling, increasing endothelial permeability, and promoting vascular inflammation, social disconnection may create a proatherogenic environment that precedes the development of carotid plaque, coronary calcification, and other manifestations of subclinical atherosclerosis.

## Social Isolation, Loneliness and Subclinical Atherosclerosis

Clinical cardiovascular events are typically preceded by a prolonged period of asymptomatic vascular disease. Subclinical atherosclerosis can be detected using imaging modalities such as carotid IMT, carotid plaque, CAC, coronary computed tomography angiography, and measures of vascular stiffness. These markers provide an opportunity to investigate whether social isolation and loneliness contribute to early vascular injury before the onset of overt cardiovascular disease. Composite measures of subclinical atherosclerosis and arteriosclerosis may serve as useful biomarkers for characterizing vascular pathways that contribute to the development of cardiovascular and neurovascular diseases, including coronary heart disease, stroke, and dementia [69].

Despite robust evidence linking social isolation and loneliness to cardiovascular morbidity and mortality, relatively few studies have directly examined imaging markers of subclinical vascular disease (Table 1). Nevertheless, the available evidence suggests that adverse social experiences may be associated with both carotid and coronary manifestations of subclinical atherosclerosis.

**Table 1:** Studies investigating associations between social isolation, loneliness, and related social factors with subclinical vascular disease

Study	Study design	Participants	Social factor assessed	Vascular phenotype	Main findings
Helminen et al. 1995 [70]	Cross-sectional	212 Finnish men aged 50–60 years	Living alone (conjugal status)	carotid IMT	Men living alone had greater carotid IMT than cohabiting men
Knox et al. 2000 [71]	Cross-sectional	> 4,600 participants from the NHLBI Family Heart Study	Hostility and social support	Carotid atherosclerotic lesions	Greater social support was associated with lower odds of carotid lesions, particularly among women with high familial CHD risk
Joo et al. 2018 [72]	Cross-sectional	1,384 adults at high cardiovascular risk	Social network betweenness	CAC	Lower social network betweenness was independently associated with greater CAC burden, whereas network size was not.
Djekic et al. 2020 [73]	Cross-sectional	1,067 middle-aged adults (SCAPIS)	Social support	CAC	Lack of social support was associated with subclinical coronary artery disease in women
Jakubowski et al. 2022 [74]	Cross-sectional	290 women aged 40–60 years	Self-silencing in intimate relationships	Carotid IMT and plaque	Higher self-silencing was associated with greater carotid plaque burden
Nordin et al. 2025 [75]	Cross-sectional	884 healthy middle-aged adults	Emotional support	Carotid IMT and plaque	Low emotional support was associated with carotid plaque formation.
Arcidiacono et al., 2023 [76]	Cross-sectional	Community-based adults	Social isolation and appraisal support	Carotid-femoral pulse wave velocity	Social isolation and low appraisal support were associated with greater arterial stiffness
Sara et al. 2025 [67]	Cross-sectional	312 adults	Social isolation	Peripheral endothelial function	Greater social isolation was independently associated with endothelial dysfunction
Baumer et al. 2025 [68]	Cross-sectional	Community-based cohort	Loneliness	Endothelial barrier integrity	Loneliness was associated with endothelial dysfunction and elevated soluble VE-cadherin concentrations

*CAC *coronary artery calcium, *CHD *coronary heart disease, *IMT *intima-media thickness, *VE *vascular endothelial

### Carotid Atherosclerosis

Early evidence linking social relationships to subclinical atherosclerosis emerged from studies of the carotid arteries. In a cross-sectional study of Finnish middle-aged men, living alone was independently associated with greater carotid IMT, suggesting that conjugal circumstances may influence the development of subclinical atherosclerosis beyond traditional cardiovascular risk factors [70]. Similarly, analyses from the NHLBI Family Heart Study demonstrated that greater social support was associated with lower odds of carotid lesions, particularly among women with a familial predisposition to coronary heart disease [71].

More recent investigations have expanded these observations. In a cohort of midlife women, higher levels of self-silencing in intimate relationships were associated with greater carotid plaque burden independent of traditional cardiovascular risk factors [74]. Likewise, low emotional support was associated with increased carotid plaque formation in healthy middle-aged adults [75]. Collectively, these findings suggest that adverse social experiences and poor social support may contribute to structural changes within the carotid arterial wall long before the development of symptomatic cardiovascular disease.

### Coronary Atherosclerosis

Evidence linking social factors to coronary atherosclerosis has primarily been derived from studies using CAC, an established marker of coronary plaque burden. In the Korean Social Life, Health and Aging Project, lower social network betweenness, reflecting limited bridging connections across social groups, was independently associated with greater CAC burden, whereas network size itself was not [72]. These findings suggest that the structure and quality of social networks may be more important than the number of social contacts.

Additional evidence was provided by the Swedish CArdioPulmonary bioImage Study, in which lack of social support was associated with subclinical coronary artery disease in middle-aged women as assessed by CAC imaging [73]. Importantly, the association was observed despite adjustment for conventional cardiovascular risk factors, supporting a potential role for social relationships in early coronary atherosclerotic processes.

### Endothelial Dysfunction and Vascular Aging

In addition to structural markers of atherosclerosis, emerging evidence suggests that social isolation may contribute to vascular dysfunction and accelerated vascular aging. Endothelial dysfunction is widely regarded as an early manifestation of atherosclerosis and frequently precedes detectable plaque formation.

Recent clinical evidence demonstrated that social isolation was independently associated with peripheral endothelial dysfunction, as assessed by reactive hyperemia index measurements, even after adjustment for traditional cardiovascular risk factors [67]. Similarly, loneliness has been associated with increased circulating concentrations of soluble vascular endothelial cadherin, suggesting impaired endothelial barrier integrity and enhanced vascular permeability [68].

Beyond endothelial dysfunction, social isolation and low appraisal social support have been associated with increased carotid–femoral pulse wave velocity, indicating greater arterial stiffness and accelerated vascular aging [76]. Although arterial stiffness reflects arteriosclerotic remodeling rather than atherosclerotic plaque formation, both processes contribute to cardiovascular risk and may represent complementary manifestations of adverse vascular aging.

## Evidence from Animal Models

Animal studies provide important support for a causal relationship between adverse social environments and atherosclerosis. Unlike human observational studies, experimental animal models allow direct manipulation of social conditions while minimizing confounding influences. Detailed discussions of animal models of social isolation and cardiovascular disease have been published previously [4, 77].

Early studies in cynomolgus monkeys demonstrated that social stress and social deprivation accelerate coronary artery atherosclerosis independently of plasma lipid concentrations [78, 79]. Similar findings have subsequently been reported in Watanabe heritable hyperlipidemic rabbits and apolipoprotein E-deficient mice, in which social isolation increased atherosclerotic lesion development compared with animals maintained in affiliative social environments [80–82]. These observations suggest that adverse social experiences can directly influence atherogenesis rather than simply acting through traditional cardiovascular risk factors.

The mechanisms underlying accelerated atherosclerosis in socially isolated animals are incompletely understood but appear to involve many of the same pathways implicated in humans, including neuroendocrine activation, autonomic dysregulation, inflammation, oxidative stress, and endothelial dysfunction [58, 80, 83]. Importantly, social isolation in animal models has been associated with increased vascular oxidative stress, enhanced inflammatory signaling, and impaired vascular function, all of which are recognized drivers of atherogenesis.

Taken together, the consistency of findings across non-human primates, rabbits, and genetically modified mouse models provides compelling evidence that adverse social environments can directly accelerate atherosclerotic lesion development. These experimental findings complement observations from human studies and support a biological framework in which social isolation and loneliness contribute to subclinical vascular disease through conserved neuroendocrine, inflammatory, and vascular mechanisms.

## Limitations

Several limitations of the current evidence should be acknowledged. First, most available studies are cross-sectional, limiting causal inference and making it difficult to determine whether social isolation and loneliness directly contribute to the development of subclinical atherosclerosis or merely coexist with other cardiovascular risk factors. Second, considerable heterogeneity exists in the assessment of social connectedness. Studies have variably examined loneliness, social isolation, social support, marital status, and social network characteristics, which represent related but distinct constructs and are often not directly comparable. Third, residual confounding by socioeconomic status, depression, lifestyle behaviors, and healthcare access remains difficult to exclude despite multivariable adjustment in many studies. Finally, relatively few investigations have evaluated imaging-based markers of subclinical atherosclerosis, and available studies differ substantially in study populations, vascular imaging modalities, and outcome definitions, limiting direct comparisons across studies.

## Knowledge Gaps

Despite growing evidence linking social isolation and loneliness to cardiovascular disease, important gaps remain regarding their role in subclinical atherosclerosis. First, the current evidence base is relatively limited and consists predominantly of cross-sectional studies, restricting causal inference. Longitudinal studies incorporating repeated assessments of social connectedness and vascular imaging are needed to determine whether social isolation and loneliness contribute to the initiation and progression of atherosclerosis.

Second, substantial heterogeneity exists in the assessment of social exposures. Studies have variably examined loneliness, social isolation, social support, marital status, and social network characteristics. Although related, these constructs represent distinct dimensions of social connectedness and should not be considered interchangeable. Greater standardization of exposure measures would improve comparability across studies and facilitate future meta-analyses.

Third, relatively few investigations have examined imaging-based markers of subclinical vascular disease. While associations with cardiovascular events are well established, evidence linking social disconnection to carotid plaque, carotid IMT, CAC, and coronary computed tomography angiography remains limited. Large prospective cohorts incorporating contemporary vascular imaging techniques are therefore needed.

Important mechanistic questions also remain unresolved. Although neuroendocrine activation, autonomic dysregulation, inflammation, oxidative stress, and endothelial dysfunction have all been implicated, their relative contributions to atherogenesis are unclear.

## Future Directions

Large prospective cohorts incorporating contemporary vascular imaging techniques are needed to clarify the relationship between social isolation, loneliness, and subclinical atherosclerosis. Recent transcriptomic and proteomic studies offer promising opportunities to identify molecular pathways through which adverse social experiences become biologically embedded and influence vascular disease. Emerging evidence also suggests that biomarkers of biological aging may provide novel insights into the mechanisms linking social disconnection with vascular aging and atherosclerosis.

Although recent systematic reviews and meta-analyses demonstrate that psychosocial, behavioral, and community-based interventions can achieve modest reductions in loneliness and social isolation [84, 85], evidence that these interventions improve vascular or cardiovascular outcomes is currently lacking. Future longitudinal studies and randomized trials incorporating vascular imaging and biological aging biomarkers are needed to determine whether reducing social isolation and loneliness can modify vascular aging, slow the progression of subclinical atherosclerosis, and improve cardiovascular outcomes.

## Clinical Implications

Evidence from prospective cohort studies suggests that the cardiovascular consequences of social disconnection extend beyond subclinical vascular abnormalities. In the Multi-Ethnic Study of Atherosclerosis (MESA), greater perceived informational support was associated with a lower risk of incident cardiovascular disease across diverse racial and ethnic groups [86]. Similarly, a recent longitudinal study demonstrated that both social isolation and loneliness were associated with an increased risk of coronary heart disease [87]. Together with evidence linking social disconnection to endothelial dysfunction, carotid plaque, and coronary artery calcium, these findings support the concept that social relationships may influence cardiovascular health across the entire disease continuum, from early vascular injury to overt clinical cardiovascular events.

## Conclusion

Accumulating evidence indicates that social isolation and loneliness are associated with endothelial dysfunction, vascular aging, and imaging markers of subclinical atherosclerosis, including carotid plaque, carotid intima-media thickness, and coronary artery calcium. Experimental animal studies further support a causal relationship by demonstrating that adverse social environments accelerate atherogenesis through interconnected neuroendocrine, autonomic, inflammatory, oxidative, and endothelial pathways. Recent transcriptomic, proteomic, and biological aging studies further strengthen the biological plausibility of these associations by identifying molecular signatures linking chronic social disconnection with cardiovascular risk.

Although the available evidence remains limited and is predominantly observational, current findings support a biologically plausible association between social isolation, loneliness, and early atherosclerosis. At present, the evidence is insufficient to support routine incorporation of social isolation or loneliness into cardiovascular risk prediction or management strategies.

## Key References

- Cene CW, Beckie TM, Sims M, Suglia SF, Aggarwal B, Moise N, et al. Effects of Objective and Perceived Social Isolation on Cardiovascular and Brain Health: A Scientific Statement From the American Heart Association. J Am Heart Assoc. 2022;11(16):e026493. 10.1161/JAHA.122.026493.
  - ⚬ Established social isolation and loneliness as emerging cardiovascular and brain health risk factors and provided a comprehensive framework linking social disconnection to cardiovascular disease.
- Ko S, Anzai A, Liu X, Kinouchi K, Yamanoi K, Torimitsu T, et al. Social Bonds Retain Oxytocin-Mediated Brain-Liver Axis to Retard Atherosclerosis. Circ Res. 2025;136(1):78–90. 10.1161/CIRCRESAHA.124.324638.
  - ⚬ Provided compelling mechanistic evidence that stable social bonds directly attenuate atherosclerosis through an oxytocin-mediated brain-liver axis.
- Shen C, Zhang R, Yu J, Sahakian BJ, Cheng W, Feng J. Plasma proteomic signatures of social isolation and loneliness associated with morbidity and mortality. Nat Hum Behav. 2025;9(3):569 − 83. 10.1038/s41562-024-02078-1.
  - ⚬ Identified molecular signatures associated with social isolation and loneliness and linked them to future cardiovascular and cardiometabolic disease, providing important mechanistic insights.
- Wang R, Yin X, Chen Y, Wang X, Shi M, Zhang Y. Loneliness, social isolation, and biological aging: Evidence from two large population-based cohort studies. J Affect Disord. 2026;406:121690. 10.1016/j.jad.2026.121690.
  - ⚬ Identified accelerated biological aging as a potential mechanism linking loneliness and social isolation with cardiovascular disease, introducing an important conceptual framework connecting psychosocial adversity with vascular aging.
- Manzato M, Kalhor P, Hamidabad NM, Nogami K, Lerman LO, Lopez-Jimenez F, et al. Persistent social connection over time is associated with attenuated biological ageing assessed by AI-enabled electrocardiography. Eur J Prev Cardiol. 2026. 10.1093/eurjpc/zwag212.
  - ⚬ Demonstrated that persistent social connectedness is associated with younger AI-derived electrocardiographic age, providing novel evidence linking long-term social relationships with cardiovascular biological aging.
- Sara JDS, Hamidabad NM, Rajai N, Breitinger S, Inojosa BM, Lerman LO, et al. Social Isolation Is Associated With Peripheral Endothelial Dysfunction. Mayo Clin Proc. 2025;100(12):2104-14. 10.1016/j.mayocp.2025.07.018.
  - ⚬ Demonstrated an independent association between social isolation and endothelial dysfunction, supporting a potential vascular mechanism linking social disconnection to atherosclerosis.
- Baumer Y, Tirado BA, Ortiz-Whittingham LR, Baez AS, Gutierrez-Huerta CA, Mendelsohn LG, et al. Loneliness associates with endothelial dysfunction in a community-based cohort: a pilot study and translational approach. NPJ Cardiovasc Health. 2025;2:28. doi: 10.1038/s44325-025-00059-5.
  - ⚬ Linked loneliness to endothelial dysfunction and impaired endothelial barrier integrity, suggesting novel pathways through which adverse social experiences may promote vascular disease.
- Hughes TM, Tanley J, Chen H, Schaich CL, Yeboah J, Espeland MA, et al. Subclinical vascular composites predict clinical cardiovascular disease, stroke, and dementia: The Multi-Ethnic Study of Atherosclerosis (MESA). Atherosclerosis. 2024;392:117521. 10.1016/j.atherosclerosis.2024.117521.
  - ⚬ Highlighted the value of composite measures of subclinical vascular disease as predictors of future cardiovascular and neurological outcomes and provides an important conceptual framework for studies of vascular aging and atherosclerosis.
- Nordin S, Norberg M, Braf I, Johansson H, Lindahl B, Lindvall K, et al. Associations between emotional support and cardiovascular risk factors and subclinical atherosclerosis in middle-age. Psychol Health. 2025;40(6):997–1011. 10.1080/08870446.2023.2286296.
  - ⚬ Provided direct evidence that low emotional support is associated with carotid plaque formation, strengthening the limited literature connecting social factors to imaging-defined subclinical atherosclerosis.
- Lasgaard M, Qualter P, Lovschall C, Laustsen LM, Lim MH, Sjol SE, et al. Are loneliness interventions effective for reducing loneliness? A meta-analytic review of 280 studies. Am Psychol. 2026;81(1):36–52. 10.1037/amp0001578.
  - ⚬ Synthesized evidence from 280 intervention studies and demonstrates that loneliness interventions produce significant, although generally modest, reductions in loneliness.
- Bergstrasser J, Schmahl T, Steinhauser J, Goetz K. Interventions against loneliness and social isolation in older adults- a systematic review. BMC Public Health. 2026;26(1). 10.1186/s12889-026-27683-9.
  - ⚬ Summarized psychosocial, behavioral, and community-based interventions targeting loneliness and social isolation in older adults and highlights the need for studies evaluating cardiovascular and vascular outcomes.

## Data Availability

No datasets were generated or analysed during the current study.

## Abbreviations

CAC: coronary artery calcium
CTRA: conserved transcriptional response to adversity
eNOS: endothelial nitric oxide synthase
HF-HRV: high-frequency heart rate variability
HPA: hypothalamic-pituitary-adrenal
IMT: intima-media thickness
